# Neurturin enhances the recovery of erectile function following bilateral cavernous nerve crush injury in the rat

**DOI:** 10.1186/1749-7221-2-5

**Published:** 2007-03-06

**Authors:** Anthony J Bella, Thomas M Fandel, Kavirach Tantiwongse, William O Brant, Robert D Klein, Carlos A Garcia, Tom F Lue

**Affiliations:** 1 Knuppe Molecular Urology Laboratory and Department of Urology, University of California, San Francisco, USA; 2 Rinat Neuroscience, South San Francisco, USA

## Abstract

**Background:**

The molecular mechanisms responsible for the survival and preservation of function for adult parasympathetic ganglion neurons following injury remain incompletely understood. However, advances in the neurobiology of growth factors, neural development, and prevention of cell death have led to a surge of clinical interest for protective and regenerative neuromodulatory strategies, as surgical therapies for prostate, bladder, and colorectal cancers often result in neuronal axotomy and debilitating loss of sexual function or continence. *In vitro *studies have identified neurturin, a glial cell line-derived neurotrophic factor, as a neuromodulator for pelvic cholinergic neurons. We present the first *in vivo *report of the effects of neurturin upon the recovery of erectile function following bilateral cavernous nerve crush injury in the rat.

**Methods:**

In these experiments, groups (n = 8 each) consisted of uninjured controls and animals treated with injection of albumin (blinded crush control group), extended release neurotrophin-4 or neurturin to the site of cavernous nerve crush injury (100 μg per animal). After 5 weeks, recovery of erectile function (treatment effect) was assessed by cavernous nerve electrostimulation and peak aortic pressures were measured. Investigators were unblinded to specific treatments after statistical analyses were completed.

**Results:**

Erectile dysfunction was not observed in the sham group (mean maximal intracavernous pressure [ICP] increase of 117.5 ± 7.3 cmH_2_O), whereas nerve injury and albumin treatment (control) produced a significant reduction in ICP elevation of 40.0 ± 6.3 cmH_2_O. Neurturin facilitated the preservation of erectile function, with an ICP increase of 55% at 62.0 ± 9.2 cmH_2_O (p < 0.05 vs control). Extended release neurotrophin-4 did not significantly enhance recovery of erectile function with an ICP change of 46.9 ± 9.6. Peak aortic blood pressures did not differ between groups. No significant pre- and post-treatment weight differences were observed between control, neurotrophin-4 and neurturin cohorts. All animals tolerated the five-week treatment course.

**Conclusion:**

Treatment with neurturin at the site of cavernous nerve crush injury facilitates recovery of erectile function. Results support further investigation of neurturin as a neuroprotective and/or neuroregenerative agent facilitating functional recovery after cavernous or other pelvic autonomic nerve injuries.

## Background

Urinary incontinence and erectile dysfunction remain a common cause of debilitating post-operative morbidity for a significant proportion of patients undergoing radical therapies for prostate, bladder, and colorectal cancers, as pelvic autonomic neurons are inadvertently axotomized, lacerated, or stretched at time of surgery [1]. For example, contemporary series report that the probability of erectile dysfunction following radical prostatectomy for clinically localized cancer of the prostate is 30–80% at 24 months. Despite advances in surgical technique, most men demonstrate compromised erectile function (incomplete, delayed, or lack of post-surgical potency) as varying degrees of cavernous nerve damage occur even with bilateral nerve-sparing procedures [2].

The emerging concept of neuromodulatory therapy recognizes that although the peripheral nervous system demonstrates an intrinsic ability to regenerate after injury, this innate response is somewhat limited and does not usually allow for a full recovery of function [3]. Accumulating evidence suggests that a return to potency following injury to the cavernous nerves is partially dependent upon axonal regeneration in the remaining neural tissues and several treatment strategies offering the potential to facilitate recovery are currently under investigation in animal models, including neurotrophins, immunophilin ligands, phosphodiesterase-5 inhibitors, and embryonic stem cells [1,4-6]. Collateral sprouting of axons occurs acutely following injury to adult peripheral neurons and growth cones target local environments supportive of regeneration. Molecular mechanisms of this process remain incompletely understood for parasympathetic neurons, as research is often hampered by difficulties selectively injuring these neurons, which are often found in close proximity or within their target organs [3]. Glial cell line-derived neurotrophic factors, including glial cell line-derived neurotrophic factor (GDNF), neurturin (NTN), persephin, and artemin represent a class of novel agents with neuroprotective and neuroregenerative properties [7]. The retrograde axonal transport mechanism of motor neurons has previously been exploited to deliver the gene encoding GDNF into the central nervous system, providing trophic support following injury [8]. NTN and GDNF have also been shown to promote survival and maintainence of cranial parasympathetic neurons via a Ret receptor tyrosine-kinase signalling component and a glycosylphosphatidylinositol-anchored GDNF family receptor α (GFRα) protein receptor complex [9]. *In vitro *studies of neurturin have demonstrated stimulation of parasympathetic neurite extension from sacral ganglia tissue cultures via the PI3-kinase pathway and suggest NTN acts as a target-derived survival and/or neuritogenic factor for penile erection-inducing postganglionic neurons via a neurotrophic signaling mechanism distinct from other parasympathetic neurons [10-12]. To date, functional improvements secondary to neurturin treatment have not been tested. In this study, the *in vivo *neuromodulatory effects of neurturin upon the recovery of erectile function following bilateral cavernous nerve crush injury are demonstrated using a rat model of neurogenic impotence.

## Methods

### Purification of neurturin

Recombinant rat neurturin (NTN) was expressed in *E. coli *as an inclusion body. Cell lysis was performed on a micro-fluidizer, repeated, and inclusion bodies were solubilized in 6 M guanidine-HCL, 0.1 M sodium sulfite, 0.01 M sodium terathionate and 0.02 M Tris pH 8.0 for 4 hours at 25°C. Separation of solubilized inclusion body rat NTN was achieved by centrifugation at 7,000 rpm for 1 hour, which was dialyzed in 4 M guanidine-HCL, 1 mM imidazole, and 0.01 M phosphate (pH 7.2). Unfolded rat NTN was then purified on an affinity nickle charge resin Ni-NTA superflow column (Qiagen Inc, Valencia, California, USA). Solubilized rat NTN was washed with 10× (ten times) column volume of 10 mM imidazole, and eluted with 0.4 M imidazole. Rat NTN fractions were exchanged in pre-refolding buffer containing 4 M urea, 0.1 M phosphate, 10% glycerol, 0.02 M glycine, and 0.02 M Tris pH 8.2. The refolding reaction was carried out by diluting rat NTN 10× in 3 M urea, 15% glycerol, 0.075 M phosphate, 0.3 M NaCL, 0.02 M glycine, 2 mM cysteine, and 0.02 M Tris pH 8.2, which was left incubating at 4°C for 48 hours. Di-filtration was performed and refolded rat NTN was formulated in 0.2 M sodium acetate pH 3.8. Refolded rat NTN was further purified on Toyopearl 650 M-phenyl sepharose HIC media (Tosoh Corp, Tokyo, Japan). Rat NTN was then loaded in 0.2 M sodium acetate and 0.750 M NaCL. A 10× column volume wash was performed in 1 M NaCL, followed by elution of rat NTN in HIC media with 0.2 M sodium acetate. Stripping of unfolded rat NTN and contamination was achieved by adding 25% ETOH to the HIC media. Finally, refolded rat NTN was formulated into 10 mM sodium acetate pH 3.8.

### Functional studies

Thirty-two male Sprague-Dawley rats (3 months old, 250–350 g) were randomly divided into four groups, each containing eight animals. Control animals received a sham operation only (identification of the cavernous nerves bilaterally). The remaining 24 animals were divided into 3 treatment cohorts (Groups A, B, and C). Animals in the treatment groups underwent a bilateral cavernous nerve crush injury, followed by direct injection of either albumen (blinded control group), extended release NT-4 or neurturin (dose of 100 ug per animal; microspheres suspended in phosphate buffered solution) to the site of injury. All animal experiments were approved by the local ethical committee for experimentation (University of California, San Francisco, Institutional and Animal Care Use Committee) and complied with National Institutes of Health (NIH) regulations for the care and use of laboratory animals.

Animals were anesthetized for surgical procedures using intraperitoneal ketamine (100 mg/kg) and xylazine (10 mg/kg) and kept isothermic on a heated pad. After the animal was shaved, a lower midline abdominal incision exposed the prostate gland and the cavernous nerves and major pelvic ganglia (MPG) were identified bilaterally. No additional pelvic surgical manipulation was performed in the control group. In groups A, B, and C, the cavernous nerves were carefully isolated and the crush injury induced using a surgical needle driver at a constant 'one-click' pressure for 2 minutes per side. The abdominal wall was subsequently closed in two layers.

At 5 weeks, erectile function was assessed by measuring maximal intracavernous pressure (ICP) upon direct cavernous nerve electrostimulation. The cavernous nerves were isolated via a repeat midline abdominal incision and the crura of the penis was identified. A 23-gauge butterfly needle with 250 U/ml heparin solution was inserted into the penile crus and connected to polyethylene-50 tubing for ICP measurement. A bipolar stainless steel hook electrode (2 mm diameter probes separated by 1 mm) stimulated the cavernous nerves. Monophasic rectangular pulses were generated by a computer with a custom-built constant current amplifier. The stimulus parameters were 1.5 mA, 20 Hz, pulse width 0.2 ms, and duration 50 s. Each cavernous nerve was stimulated separately, ICP measured using LabVIEW 4.0 software (National Instruments, Austin, Texas), and mean maximal right and left ICPs determined for each rat. Systemic blood pressure was measured prior to terminating the procedure using a butterfly needle inserted into the aorta.

The data were first analyzed by non-repeated measures ANOVA with significance considered at p < 0.05. If the difference was significant, Student Newman-Keuls test was performed. All results were expressed as the mean ± SEM. Animal weights prior to and following treatment were compared. If an adverse event occurred, the cause of mortality or early cessation of therapy (eg. weight loss, visible lesions/tumor) and timepoint was noted. Investigators were unblinded after statistical analyses were completed.

## Results

**Figure 1:**
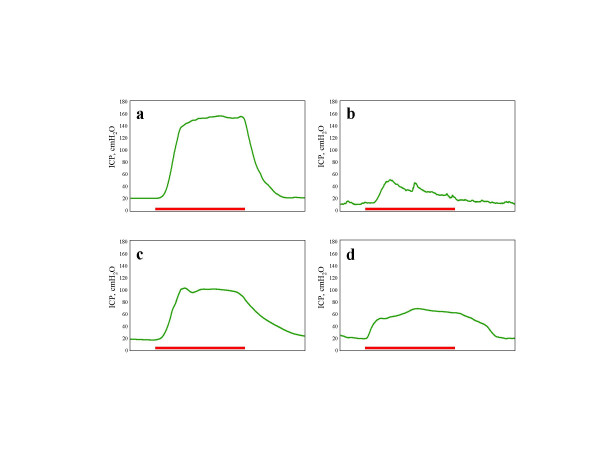
Examples of intracavernous pressure changes after electrostimulation of the cavernous nerves at 5 weeks. (a) Sham (uninjured) group, (b) albumin (crush control), (c) neurturin treatment, and (d) neurotrophin-4. The x-axis is in seconds, and the red line represents 50s of stimulation.

To evaluate recovery of erectile function, the increase in maximal intracavernous pressure (which correlates to penile rigidity in men) was measured (Figure 1). Erectile dysfunction was not observed in the uninjured control group, which served to establish a baseline normal erectile response to stimulation. The mean maximal intracavernous pressure [ICP] increase observed was 117.5 ± 7.3 cmH2O. The blinded control group, which was treated with albumin only, demonstrated a significant reduction for increased ICP of 40.0 ± 6.3 cmH2O, consistent with a state of erectile dysfunction. Neurturin facilitated the preservation of erectile function, with a mean ICP increase of 55%. The increase of 62.0 ± 9.2 cmH2O was statistically significant (p < 0.05 vs control). Extended release neurotrophin-4 did not significantly enhance recovery of erectile function with ICP changes of 46.9 ± 9.6 (Table 1). No statistically significant differences were observed between all groups for peak aortic blood pressure or weight gain. There were no animal deaths or incomplete treatments in this study.

**Table 1:** Intracavernous pressure increase in response to electrostimulation five weeks following bilateral cavernous nerve crush injury.

Group	Cavernous Pressure Increase (mean cm H_2_O ± SEM)
a. Sham (uninjured)	117.5 ± 7.3
b. Albumin (blinded crush control)	40.0 ± 6.3^###^
c. Neurturin (100 ug)	62.0 ± 9.2**
d. Extended-release NT-4 (100 ug)	46.9 ± 9.6

^###^Versus Sham p < 0.001

**Versus Control p < 0.05

## Discussion

A clear clinical need for the development of therapeutic neuromodulatory interventions has been defined as both sympathetic and parasympathetic pelvic innervation is at high risk of injury during surgery or radiation therapy for prostate, bladder, and colorectal malignancies. Penile erection, controlled by adrenergic, cholinergic, and nonadrenergic noncholinergic (NANC) neuroeffectors carried in the cavernous nerves, is often compromised by these treatments, and subsequent patient quality-of-life diminished [4]. Despite advances in operative technique, the probability of a man undergoing open radical retropubic prostatectomy for clinically localized disease and achieving cancer-control, continence and potency is approximately 60% at 24 months [12]. Neurturin, which is expressed in peripheral neuronal targets including the penis, has demonstrated key neuromodulatory properties including retrograde transport from the periphery to cell body, enhancement of neuronal survival and promotion of neurite outgrowth [13-15]. In this study, we demonstrate neurturin's ability to confer an *in vivo *advantage for the functional recovery of erectile function following cavernous nerve injury.

Various methods of inducing injury to the cavernous nerves are described in literature and include nerve transection, cryoablation, crush and partial excision [16]. We prefer to use a controlled bilateral nerve crush technique, as significant but reversible damage to penile innervation occurs and allows for the evaluation of functional recovery. Advantages of this technique include simplicity, reliability, and reproducibility, albeit the relationship to surgical trauma incurred by prostatectomy is inexact as the prostate itself is not removed [17]. Because NOS-containing nerves and neurons are the principal sites where the erection-inducing neurotransmitter nitric oxide (NO) is synthesized, their loss after nerve injury is therefore chiefly responsible for the development of ED. Using this animal model of neurogenic ED, we have previously demonstrated a significant loss of nitric oxide syntheses (NOS)-containing nerve fibers and neurons in the corpora cavernosa and in the major pelvic ganglia (MPG) respectively, within one month of bilateral cavernous nerve crush injury [18].

Neurturin applied directly to the area of injury facilitated the preservation of erectile function as compared to untreated control animals and extended release neurotrophin-4. The primary outcome measure, mean intracavernous pressure increase, has been used extensively as the measure of penile rigidity (function) in a wide variety of ED animal models, and is a unifying factor for defining response in the treatment of erectile dysfunction in humans [19]. In a recently reported study investigating the relationship between mean arterial pressure and ICP, MacKenzie et al demonstrated that changes in ICP values were adversely effected only when mean arterial pressure (MAP) fell below 70 mmHg (regardless of the cause) [20]. Aortic pressures following determination of ICP did not differ between groups and each animal demonstrated values of 100 mmHg or greater. Therefore, a carotid artery catheter was not placed to monitor arterial pressure concurrently with cavernous nerve stimulation as performed in the past, minimizing undue operative morbidity and physiologic stress on the rat as recommended by our Institutional Review Board. In addition, our preference is to observe the wave form and the ICP change rather than the ratio of ICP/BP; hypertensive patients can have abnormal ICP/MAP ratios but sufficient penile rigidity (with intracavernous pressures exceeding 100 mmHg) and would not be labelled as impotent.

Following injury, compensatory and regenerative sprouting of penile-projecting nerve fibres is likely driven by, and dependent upon, various neurotrophic factors including NTN, which is synthesized in urogenital tissues including the penis and may also be secreted by glial cells within the ganglion or glia associated with the injured axon(s) [3]. Known receptors for neurturin include the GDNF family receptors α1, α2 (predominant), and α4, and have been identified in the major pelvic ganglion [21]. Pelvic parasympathetic ganglion neurons respond to axotomy by altering expression of NTN receptors; altered glial secretions or glial coupling represent a complimentary second mechanism of adapative signalling in early phases of regeneration [3]. As penis-projecting pelvic neurons express neuronal nitric oxide (nNOS) and GFRα2, accumulating tissue culture, cell-line, *in vivo *signalling, and with this report functional evidence, suggests that neurturin plays a role in regeneration, as well as maintainence of adult parasympathetic neurons [11,22]. Given the limitations of this pilot study, including unknown optimal dosing or site of NTN delivery (crush site versus major pelvic ganglion or penis), and an incomplete understanding of the neurobiology of cavernous nerve and neurturin interaction, results are encouraging and warrant further study of NTN in this role. Following a similar course to our investigations of brain-derived nerve growth factor and its role in cavernous nerve response to injury, we plan to focus upon identifying the primary molecular signalling pathway(s), concentration-dependent effects, and pattern(s) of endogenous neurturin release in an effect to better delineate its neuroregenerative or neuroprotective properties [23,24].

A growing body of literature suggests neurturin may represent a promising therapeutic agent for both central and peripheral neurologic diseases states, enhancing survival, differentiation, and regeneration of neurons alone or synergistically with other molecules. In addition to traumatic injury, neurogenic impotence is often associated with diseases related to sensory and/or peripheral neuropathy such as diabetes mellitus [1]. As penile tissues are known to express mRNA transcripts for at least 10 neurotrophic factors, treatment strategies utilizing neurturin and these neuromodulators alone or in combination may represent future approaches to alleviate ED caused by injury, neurological or vascular changes [25,26]. From a broader perspective, elucidating the mechanisms by which neurturin enhances peripheral nerve repair and functional recovery may translate into clinical applications for such diverse conditions as recurrent laryngeal nerve and brachial plexus injuries, iatrogenic neuropraxias, or urinary incontinence secondary to hysterectomy.

## Conclusion

Treatment with neurturin at the site of cavernous nerve crush injury facilitates recovery of erectile function in a bilateral cavernous nerve crush injury model of erectile dysfunction in the rat. Results support further investigation of neurturin as a neuroprotective and/or neuroregenerative agent following cavernous or other pelvic autonomic nerve injuries.

## Competing interests

AJB, TMF, KT, and WOB declare no competing interests. RDK and CAG were employees of Rinat Neuroscience at the time of this study. TFL received funding for this study from Rinat Neuroscience.

## Authors' contributions

AJB designed the study, performed crush injury (CI) surgeries, measurement of intracavernous pressure response of electrostimulation (ICP), and drafted the manuscript. TF and KT helped perform CI and ICP surgeries. WOB participated in study design, drafting of the manuscript, and performed statistical analyses. RDK and CAG synthesized neurturin, extended-release neurotrophin-4 and the blinded control, and contributed the NTN purification protocol to the manuscript. TFL conceived the study, participated in its design and drafting of the manuscript.
